# The differential effect of psychopathy on active and bystander trolling behaviors: the role of dark tetrad traits and lower agreeableness

**DOI:** 10.1038/s41598-024-60203-6

**Published:** 2024-04-30

**Authors:** Anantha Ubaradka, Sanjram Premjit Khanganba

**Affiliations:** 1 Human Factors and Applied Cognition Lab, Indian Institute of Technology Indore, Indore, 453552 India; 2 Discipline of Psychology, Indian Institute of Technology Indore, Indore, 453552 India

**Keywords:** Human behaviour, Motivation

## Abstract

This study aimed to develop the Global Assessment of Active Trolling and Passive Bystanderism (GAATPB) scale and investigate the influence of personality traits on trolling behaviors. Focusing on the Dark Tetrad (DT) traits and agreeableness, the present study examined their associations and predictive utility on active trolling and passive bystanderism. Participants were recruited from social networking sites (SNSs), and eligibility criteria included active SNS usage and engagement in online interactions. A total of 797 healthy adult students participated in the study, with data from 300 used for the initial exploratory factor analysis (EFA) and the remaining 497 (*M*_*age*_ = 22.25 years, SD = 3.37) for the subsequent analyses. Results indicated a significant correlation between DT traits and agreeableness across both active trolling and passive bystanderism, revealing a shared personality profile. Hierarchical multiple regression analyses showed that narcissism, Machiavellianism, and trait sadism were predictors of active trolling, with psychopathy being the strongest predictor. However, psychopathy did not emerge as a predictor for passive bystanderism. The study also highlighted that DT traits mediated the relationship between lower agreeableness and overall trolling behavior, suggesting that trolling manifests from lower agreeableness through the instigation of callous-unemotional, manipulative, and self-centered traits inherent in DT.

## Introduction

Internet trolling (or just trolling) is an antisocial online behavior, defined as a deliberate attempt to initiate unproductive and protracted discussions on social networking sites (SNSs) or discussion forums^1,2^. However, the terms ‘troll’ and ‘trolling’ can carry multiple, often inconsistent meanings, varying with the context of usage and the intentions of the user^3,4^. When used nominally, a troll identifies an individual who perpetrates such behavior. In its verbal form, the term draws a metaphorical parallel to a ‘fishing’ technique in which bait is dragged through the water to attract fish^5^. This analogy aptly captures the essence of trolling, where the perpetrator baits and provokes others in a digital environment. Trolls engage in aggressive online interactions, akin to cyberbullying and cyberstalking^6^, sometimes without full awareness of their target or the nature of their actions^7^. They often exploit anonymity to provoke conflict and demean others, making their targets appear foolish^1,8^. Common trolling behaviors include instigating contentious arguments, social manipulation, attention-seeking, and disseminating harmful messages^1,3,5,9^. These behaviors are often driven by motives such as boredom, attention-seeking, revenge, and communal animosity^1,10^. However, research indicates that trolling can also manifest in less malevolent forms^11,12^, suggesting a need to broaden the assessment and conceptualization of trolling as a multidimensional construct.

Early studies on trolling used qualitative methods, including in-depth interviews^13^ and content analysis of online posts^5^. Buckels et al.^1^ introduced the Global Assessment of Internet Trolling (GAIT), a four-item instrument designed to empirically evaluate trolling dimensions like experience, enjoyment, and self-identification. Later, Cracker and March^9^ developed the Global Assessment of Facebook Trolling (GAFT), a variant specifically adapted for the Facebook environment. Recognizing the content validity limitations of these scales, Sest and March^14^ proposed an improved version, the Revised Global Assessment of Internet Trolling (R-GAIT), which included additional items. Although studies affirm that internet trolling encompasses diverse behaviors and motivations^5,15^, the predominant focus has been on active interaction between the perpetrator and the victim, overlooking the role of bystanders.

Bystander behaviors manifest in online environments through a range of actions, from supporting the victim to remaining passive or even reinforcing the perpetrator^16^. Consequently, bystander intervention may assume both active and passive roles, exerting influences on the victim that can be either beneficial or detrimental. Positive bystander interventions, such as directly comforting victims, can mitigate the negative impact of online misconduct^17^. Similarly, actions such as reporting incidents to authorities, can reduce aggressive content and foster positive online social norms^18^. While positive bystander intervention can be beneficial, there is a notable tendency towards passivity during online misconduct^19^. Research suggests that sustained inactivity and a lack of empathy among passive bystanders may lead them to become active perpetrators^20^. The combination of passive bystander behavior and reinforcement of perpetrators can exacerbate online misconduct and amplify victim trauma^21^. This dynamic reflects patterns seen in traditional bullying, where bystander endorsement can escalate the aggressor’s actions^22^.

Bystander intervention in online misconduct is contingent upon the perceived sense of connection with the victim and personal safety^23^. A lack of such connection may lead to bystander passivity, which could potentially evolve into active aggression. The dynamics within SNSs further complicate this scenario. Users frequently encounter misconduct on these platforms beyond their immediate social circles, facilitated by features like ‘public content visibility’ and connections with ‘friends-of-friends.’ These features, along with the tendency of SNSs to foster ‘weak tie’ relationships, can contribute to a sense of anonymity, thereby diminishing the sense of connectedness crucial for effective bystander intervention^24^. The prevalent anonymity on the internet, especially in trolling scenarios^1^, underscores the need for a careful examination of passive bystander behaviors in these contexts. While passive bystander behaviors have been explored in other forms of online misconduct, such as cyberbullying and online hate^23,25^, there is a scarcity of studies addressing this issue in the context of internet trolling.

To address this research gap, the present study aims to develop the Global Assessment of Active Trolling and Passive Bystanderism (GAATPB) scale. This scale employs a two-dimensional framework to evaluate trolling behaviors, covering both active and passive bystander components. Active trolling is defined in its conventional form as intentional provocation or harassment conducted on SNSs^1^, characterized by deliberate actions intended to distress or provoke other users^5^. On the other hand, ‘passive bystanderism’ is examined, acknowledging its catalytic role in harming the victim and reinforcing trolls within online environments. The present study defines passive bystanderism as an act of observing or consuming provocative content on SNSs without directly participating in trolling activities or inflicting harm on the target. This passive engagement may include behaviors ranging from silently viewing and enjoying such interactions to subtly endorsing them. Furthermore, it examines the role of personality characteristics on the dimensions of trolling behavior, exploring the associations and predictive utility of dark personality traits and agreeableness on active trolling and passive bystanderism.

There has been a growing emphasis on exploring dark personalities and examining how these traits correlate with and influence trolling behavior. The Dark Triad is the most commonly used model to assess these malevolent traits, encompassing narcissism, Machiavellianism, and psychopathy^26^. However, the addition of trait sadism^27^ expands the framework to the Dark Tetrad (DT). Although these personality traits overlap and share characteristics like callousness, manipulation, and apathy^27^, they also exhibit distinctive attributes^1^. Narcissism involves grandiose self-perceptions about intelligence, power, and physical appeal^28,29^, whereas Machiavellianism is associated with deceptive behaviors and social manipulation^30^. Psychopathy is characterized by impulsivity and callous-unemotionality, indicated by a lack of empathy or guilt^31^. Trait sadism, closely related to psychopathy, involves deriving pleasure from others’ suffering^32^.

Individuals exhibiting high levels of Machiavellianism and narcissism engage in behaviors detrimental to others, primarily when it serves their self-interest and objectives^33^. Research has shown that both narcissism and Machiavellianism correlate positively with the enjoyment of trolling^1^. However, it is observed that when accounting for the shared variance among DT traits, neither narcissism nor Machiavellianism significantly predicts trolling behavior^9,34,35^. Reflecting on this, Craker and March^9^ suggested that individuals with high Machiavellianism may not favor the impulsive nature of trolling, preferring controlled and calculated approaches. They also speculate that highly narcissistic individuals, due to their self-absorption, may be less inclined to exert the effort required for aggressive trolling^9^.

Conversely, there is growing evidence that psychopathy and trait sadism have the most substantial predictive utility for trolling behavior^1,9,14^. Individuals exhibiting high levels of psychopathy are predisposed to behaviors that are impulsive, violent, and antisocial^36^. Pronounced psychopathy and sadistic traits are often associated with deriving pleasure from inflicting torment, with individuals displaying the willingness to undergo challenges in such conduct^37^. This propensity aligns with the behaviors of trolls who invest time and effort to anonymously disrupt and harm others on SNSs^39,40^.

In addition to the DT, the Big Five personality traits—openness, conscientiousness, extraversion, agreeableness, and neuroticism—have also been examined in the trolling literature. Studies have identified associations between trolling and some of these traits, such as higher levels of extraversion, reduced conscientiousness, and lower agreeableness^1,34,38^. However, except for lower agreeableness, these associations demonstrate variability and are not consistently reported across the literature^39^. Lower agreeableness (often conceptualized as antagonism) is characterized by tendencies toward meanness, inconsideration, and uncooperativeness, often resulting in deviant online interpersonal behaviors^40^. For instance, individuals with lower agreeableness are found to mock others, post harmful comments^41^, or even pursue vengeful actions on SNSs^42^. Such individuals often struggle to navigate hostility and disagreement in interpersonal interactions, primarily due to reduced empathy^43^. This propensity for less empathetic, hostile, and more antagonistic interactions can extend to online environments, particularly in situations that involve witnessing online misconduct. For instance, Zhou et al.^44^ reported that among the examined Big Five personality traits, lower agreeableness was the unique trait capable of simultaneously predicting both active participation in and bystander behavior within cyberbullying. However, to our knowledge, no study has yet explored the role of lower agreeableness in relation to passive bystander behavior in the context of internet trolling.

Considering its callous nature, there is an ongoing debate regarding whether lower agreeableness constitutes the common core of dark personalities or it diverges from them^30,45^, and whether this association can be extended to a causal relationship. Many studies underscore a substantial relationship between lower agreeableness with Dark Triad^46,47^ and DT traits^48^, suggesting a considerable overlap between these constructs. According to this line of argument, the commonalities among dark personalities may fundamentally reflect the opposing pole of the agreeableness dimension. For instance, lower agreeableness emerged as a fundamental component across the subscales of the Youth Psychopathic Traits Inventory (YPI)^49^. This tool encompassed ten scales organized into three first-order factors—Grandiose/Manipulative (G/M), Callous/Unemotional (C/U), and Impulsive/Irresponsible (I/I), collectively forming a second-order factor termed ‘psychopathic personality.’ Sherman et al.^50^ found that in a sample of college students, lower agreeableness accounted for over 55% of the variance in first-order factors and 45% of the variance in second-order factors of the YPI, underscoring its critical role in the structure of psychopathic personality. This trend extends to other dark personalities as well. For instance, the variance in narcissism attributable to lower agreeableness has been reported to range from 33%^51^ to 79%^52^. Similarly, this explained variance falls between 77%^51^ and 84%^46^ for Machiavellianism, as reported in the previous studies.

While acknowledging closer associations between these traits, it is critical to observe that the theoretical foundations of agreeableness and dark personalities fundamentally differ. Agreeableness is examined as a basic personality structure, derived from lexical studies to describe all major individual differences through as few independent dimensions as possible^53^. In contrast, dark personalities such as DT traits embody a confluence of various characteristics across basic personality dimensions. This perspective implies that the examined relationship between lower agreeableness and dark personalities could be causal, where lower agreeableness might predispose individuals to DT traits, rather than the other way around. Thus, lower agreeableness may not only be necessary but also sufficient for developing certain dark personality traits, making it an antecedent in the manifestation of their motives^45,54^.

Drawing upon the research findings discussed above, lower agreeableness and DT traits have been identified as significant predictors of active trolling. However, their association with bystander behaviors remains unexplored within the context of internet trolling. To bridge this research gap, the present study investigated the relationship and predictive utility of DT traits and lower agreeableness on the propensity for active trolling and passive bystanderism. Although the actions of perpetration and bystander intervention in online misconduct diverge, they both stem from a shared underlying motive of a lack of empathy^14,55–58^. Furthermore, considering that reduced empathy is also a characteristic associated with both DT traits^59,60^ and lower agreeableness^61^, it raises an intriguing question as to whether these traits differentially influence active trolling and passive bystanderism. To address this research question, the present study hypothesized that there would be no difference between active trolling and passive bystanderism in their relationship with the personality traits (hypothesis 1), the predictive utility of these traits (hypothesis 2), and the assumed mediation of DT traits in the relationship between lower agreeableness and these trolling dimensions (hypothesis 3). Expanding on these primary hypotheses, the study further explored the specific associations of DT traits and lower agreeableness on active trolling and passive bystanderism through additional sub-hypotheses.

Firstly, this study examined the relationship between personality traits and trolling dimensions. Drawing on previous studies^1,9,14^, it proposed a significant correlation between DT traits and agreeableness, with active trolling (hypothesis 1a) and passive bystanderism (hypothesis 1b). Subsequently, it examined the predictive utility of DT traits and agreeableness on both trolling dimensions. As discussed earlier, while narcissism and Machiavellianism are found to be significantly correlated with trolling behavior, their predictive utility has not been consistently reported^1,34,39^. Addressing this issue, the study hypothesized that in addition to agreeableness, only psychopathy and trait sadism would predict active trolling (hypothesis 2a) and passive bystanderism (hypothesis 2b). Furthermore, reflecting on the precursory role of lower agreeableness in fostering DT traits^54^, which in turn emerged as a predictor of trolling behaviors, it is hypothesized that DT traits would mediate the relationship between agreeableness and both active trolling (hypothesis 3a) and passive bystanderism (hypothesis 3b).

## Methods

### Participants

The study engaged a total of 797 adult participants who were recruited via advertisements on SNSs (Facebook, Instagram, and Twitter). These advertisements contained a URL that directed potential participants to the online survey hosted on Google Forms.

Eligibility criteria included active use of at least one SNS and engagement in online social interactions, such as liking, commenting, and sharing content on these platforms. An exploratory factor analysis (EFA) was initially conducted with 300 participants to identify the dimensions of trolling behavior. Due to inadequate communality and lack of responses from the participants, an item was removed from the scale. A follow-up CFA was conducted with 497 participants (Males = 29.2%, Females = 69.21%; *M*_*age*_ = 22.25 years, SD = 3.37), and the same data was used for all subsequent analyses. Most participants identified Instagram as their preferred SNS for online activities (67.8%), followed by Facebook (10.8%). Prior to participation, written informed consent was obtained from each participant. Ethical approval for the study was granted by the Institute Human Ethics Committee of the authors’ affiliated institution, consistent with the ethical standards of the 1964 Declaration of Helsinki and its later amendments.

### Measures

#### Dark personality traits

The Dark Triad traits were assessed using the Dirty Dozen^62^, a 12-item self-report questionnaire. Participants indicated their level of agreement on a five-point scale (1 = *disagree strongly* to 5 = *agree strongly*) with statements targeting narcissism (e.g., “I tend to want others to admire me”), Machiavellianism (e.g., “I have used deceit or lied to get my way”), and psychopathy (e.g., “I tend to lack remorse”). The Dirty Dozen scale demonstrated satisfactory internal consistency on its subscales: Machiavellianism (Cronbach’s α = 0.80), narcissism (Cronbach’s α = 0.82), and psychopathy (Cronbach’s α = 0.75). Additionally, the Short Sadistic Impulse Scale^63^, comprising ten items, was employed to measure trait sadism (e.g., “people would like hurting others if they gave it a go”). The two scales were combined to yield an overall DT score (Cronbach’s α = 0.87).

#### Agreeableness

Agreeableness was measured using nine items from the Big Five Personality Inventory’s agreeableness domain^64^. Participants rated their agreement with statements (e.g., “I see myself as someone who is helpful and unselfish with others”) on a five-point scale (1 = *disagree strongly* to 5 = *agree strongly*). The scale exhibited satisfactory internal consistency (Cronbach’s α = 0.79).

#### Trolling behavior

The investigators incorporated the items from GAIT^1^ and the R-GAIT^14^ scales to develop the proposed GAATPB. All four items were retained from the original GAIT scale, and one item was incorporated from the R-GAIT (“I enjoy upsetting people on Social Networking Sites”). The scale was adapted for the Indian context, replacing slang in the original items with language more culturally appropriate to the region. For instance, “I have sent people to shock websites for the lulz” was rephrased as “I have sent comments to people on social networking sites for fun.” Additionally, three new items were specifically developed by the researchers to measure passive bystanderism. Both population and expert sampling were employed for item generation^65^. Interviews with target population members and three subject experts ensured the representativeness of items to the passive bystanderism. Subsequently, the researchers consulted colleagues for final input and integrated their feedback before administering the measure. All items were specifically designed to evaluate trolling behaviors in the context of SNSs. The initial version of the scale comprised eight items, with responses recorded on a five-point Likert scale (1 = *disagree strongly* to 5 = *agree strongly*). However, an item was excluded following the initial EFA due to insufficient communality. Detailed descriptions of the development process of the GAATPB are further elaborated in the results section.

### Data analysis

The present study utilized the Statistical Package for the Social Sciences (SPSS^®^) Version 27 and Analysis of Moment Structures (AMOS) Version 22 to analyze the data. An EFA was conducted on the pilot study data (*N* = 300) to assess the factor structure of the measuring scale. Prior to item retention, standard assumptions of the EFA, including normality, sampling accuracy, sphericity, communality, and factor loadings, were verified. Should any items fail to meet these criteria, they were removed, and a further iteration of the EFA was performed on the same dataset. Missing data was replaced using multiple imputation method in the SPSS^®^. The EFA used principal axis factoring with a direct oblimin rotation to allow for the potential correlation between factors. Subsequently, a CFA was carried out on the field study data (*N* = 497) to validate the identified factor structure using the maximum likelihood estimation.

Further, a bivariate Pearson product-moment correlation and two-step hierarchical multiple regression analyses were conducted to examine the relationship and predictive utility of DT traits and agreeableness with active trolling and passive bystanderism. Standard assumptions were evaluated before running these tests. The first step of the regression analyses involved agreeableness as the predictor variable, followed by the introduction of DT traits. The predictors were entered in an order that aligned with the previous study^66^. Further, to examine the mediating role of DT in the relationship between agreeableness and trolling dimensions, a structural equation modeling (SEM) was utilized, employing maximum likelihood estimation. Bootstrapping was set to 2000 samples, and bias-corrected 95% confidence intervals (CIs) were incorporated to enhance the robustness and accuracy of the analysis^67^.

The CFA and mediation analyses utilized various fit indices for model evaluation, including the Chi-square test, comparative fit index (CFI), goodness-of-fit index (GFI), and root mean square error of approximation (RMSEA). Adequate model fit was indicated by a nonsignificant Chi-square (*p* > 0.05), χ^2^/df < 5, CFI and GFI values exceeding 0.95, and RMSEA values below 0.08^68,69^. However, considering the sensitivity of the Chi-square test to sample size, the model fit estimation was primarily based on CFI, GFI, and RMSEA values^70^.

### Ethical statement

Each participant provided written informed consent before participating in the study. Ethical approval was secured from the Institute Human Ethics Committee of the authors’ affiliated institution, adhering to the ethical standards outlined in the 1964 Declaration of Helsinki and its subsequent amendments. Participants received non-monetary rewards as compensation for their time.

## Results

### Exploratory factor analysis

An EFA was conducted on the pilot study data (*N* = 300) for all eight items of the measuring scale. The analysis yielded insufficient communality for the item “I enjoy deliberately irritating other players while playing multiplayer games in social networking sites.” Moreover, many of the participants (47.33%) reported that they were not engaged in multiplayer games. Consequently, this item was excluded from further analyses.

The remaining seven items were subjected to another iteration of EFA on the same data. Firstly, the skewness and kurtosis values remained under the prescribed thresholds of 2 and 7, respectively, suggesting a normal distribution of the data^71^. The analysis demonstrated robust sample adequacy, evidenced by a Kaiser–Meyer–Olkin (KMO) test score of 0.78^72^. Additionally, Bartlett’s test of sphericity yielded a prominent result (χ^2^ = 714.35, *p* < 0.001), indicating the suitability of factor analysis for the dataset^72^. Communalities obtained through principal axis factoring were above the 0.40 threshold, underscoring the strength and coherence of all seven items. The selection of components for extraction was guided by the Kaiser criterion, which recommends retaining factors with eigenvalues greater than one^73^. Following these guidelines, the EFA yielded a two-factor solution for the seven items of the GAATPB scale, satisfying the eigenvalue criterion (active trolling = 3.28, passive bystanderism = 1.17). The criteria for item retention also included factor loadings above 0.50 or parallel loadings below 0.20^74^. Table 1 presents the items associated with each factor and provides the rotated component matrix of all seven items. Furthermore, the GAATPB scale exhibited adequate internal consistency, as indicated by Cronbach’s α of 0.81^75^. The two factors within the scale, active trolling (Cronbach’s α = 0.70) and passive bystanderism (Cronbach’s α = 0.82), also demonstrated satisfactory reliability values^75^.

**Table 1:** Rotated component matrix of the GAATPB scale. Rotations are converged to six iterations using principal axis factoring and direct oblimin method with Kaiser normalization.

Item number	Items	Active trolling	Passive bystanderism
Item 1	I have sent comments to people on Social Networking Sites for fun	0.54	− 0.01
Item 2	I like to troll people in forums or the comments sections of Social Networking Sites	0.76	− 0.02
Item 3	The more beautiful and purer a thing is, the more satisfying it is to corrupt	0.53	0.04
Item 4	I enjoy upsetting people on Social Networking Sites	0.60	− 0.03
Item 5	I enjoy seeing people trolling each other, though I am not directly involved	0.13	− 0.74
Item 6	I prefer following troll pages on Social Networking Sites	− 0.06	− 0.76
Item 7	I find it funny seeing others getting trolled	0.05	− 0.85

### Confirmatory factor analysis

**Figure 1:**
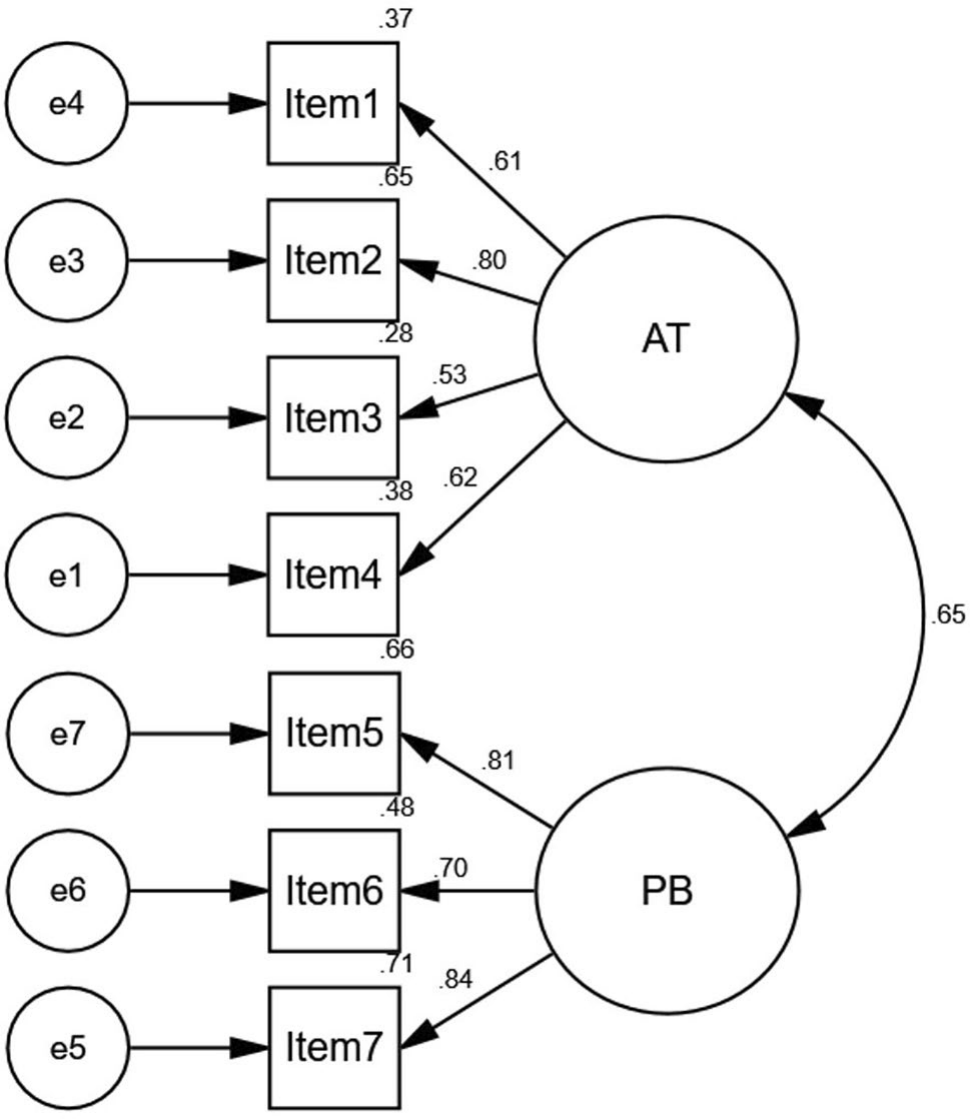
Confirmatory factor analysis of the GAATPB scale with the dimensions AT (active trolling) and PB (passive bystanderism). *N* = 497, χ^2^ = 34.34, *p* < 0.01, χ^2^/df = 2.82, CFI = 0.98, GFI = 0.98, RMSEA = 0.06.

A CFA was conducted on the field study data (*N* = 497) to ascertain the model fit for the overall sample. The results indicated (see Fig. 1) an adequate fit of the model^73^, which was corroborated by various goodness-of-fit indices (CFI = 0.98; GFI = 0.98; RMSEA = 0.06), except for the Chi-square (χ^2^ = 34.34, *p* < 0.01, χ^2^/df = 2.82).

### Correlation and hierarchical multiple regression analyses

Table 2 indicates the descriptive values and correlations between the trolling dimensions, agreeableness, and DT traits. The zero-order correlations showed significant associations between all the examined variables, supporting hypotheses 1a and 1b. Notably, agreeableness was negatively associated with all four DT traits, active trolling, and passive bystanderism. Further, DT traits were positively associated with both the trolling dimensions.

**Table 2:** Values on the descriptive and correlation analyses. *N* = 497, **p < 0.01. *AT* active trolling, *PB* passive bystanderism, *AG* agreeableness, *NA* narcissism, *MA* Machiavellianism, *PS* psychopathy, *SA* sadism.

	Mean	SD	AT	PB	AG	NA	MA	PS	SA
AT	1.90	0.72	1						
PB	2.53	1.00	0.50**	1					
AG	3.75	0.50	− 0.29**	− 0.22**	1				
NA	2.76	0.91	0.35**	0.39**	− 0.27	1			
MA	2.32	0.83	0.43**	0.41**	− 0.37**	0.52**	1		
PS	2.23	0.75	0.42**	0.33**	− 0.37**	0.39**	0.52**	1	
SA	1.76	0.43	0.38**	0.34**	− 0.38**	0.32**	0.39**	0.43**	1

Subsequent to the correlation, two-step hierarchical multiple regression analyses were conducted separately for active trolling and passive bystanderism. Prior to the analyses, standard assumptions of the regression were verified. To begin with, outliers exceeding three standard deviations were excluded to ensure data integrity. Additionally, the outcomes indicated no collinearity between the independent variables on both the trolling dimensions (VIF < 10 and tolerance > 0.1). Further, no autocorrelation was detected in the residual terms for either active trolling (Durbin–Watson value = 2.04) or passive bystanderism (Durbin–Watson value = 1.93), satisfying the criteria for independent errors. The analyses also confirmed the normal distribution of errors, homogeneity of variance, and linearity. The details of these assumptions are illustrated with suitable graphs and tables in the supplementary data.

The hierarchical regression analyses aimed to assess whether adding different variables accounted for the change in variance of the preceding predictor on the trolling dimensions (see Table 3). In the first step, agreeableness was introduced as a predictor, accounting for 8.6% of the total variance on active trolling [*R*^2^ = 0.09, *F*(1, 495) = 46.75, *p* < 0.01] and 4.6% of the total variance on passive bystanderism [*R*^2^ = 0.05, *F*(1, 495) = 25.09, *p* < 0.01], thus serving as a unique predictor for both dimensions.

**Table 3:** Values on the two-step hierarchical multiple regression analyses. *N* = 497, *p < 0.05, **p < 0.01.

Variables	Active trolling			Passive bystanderism		
	*B* (SE)	β	*t*	*B* (SE)	β	*t*
Step 1						
Constant	13.99 (0.94)		14.86**	12.55 (0.99)		12.62**
Agreeableness	− 0.19 (0.03)	− 0.29	− 6.84**	− 0.15 (0.03)	− 0.22	− 5.01**
Step 2						
Constant	2.98 (1.31)		2.28*	1.38 (1.39)		0.99
Agreeableness	− 0.04 (0.03)	− 0.06	− 1.48	0.01 (0.03)	0.01	0.01
Narcissism	0.09 (0.04)	0.11	2.35*	0.16 (0.04)	0.20	4.25**
Machiavellianism	0.16 (0.04)	0.18	3.55**	0.18 (0.05)	0.19	3.76**
Sadism	0.11 (0.03)	0.17	3.75**	0.12 (0.03)	0.17	3.65**
Psychopathy	0.18 (0.05)	0.19	4.00**	0.08 (0.05)	0.08	1.69

The second step involved adding DT traits, which significantly explained 27% of the total variance on active trolling [*R*^2^ = 0.28, *F*(5, 491) = 37.60, *p* < 0.01] and 23.6% of the total variance on passive bystanderism [*R*^2^ = 0.24, *F*(5, 491) = 31.22, *p* < 0.01]. The change of variance for both active trolling (*ΔR*^2^ = 0.19, *F*(4, 491) = 32.35, *p* < 0.01) and passive bystanderism [*ΔR*^2^ = 0.19, *F*(4, 491) = 31.22, *p* < 0.01] was statistically significant.

The results did not support hypotheses 2a and 2b, suggesting that both narcissism and Machiavellianism emerged as predictors of active trolling and passive bystanderism. Furthermore, the overall findings of the regression analyses indicated that psychopathy was the strongest predictor (β = 0.19, *p* < 0.01) of active trolling (see Table 3). On the other hand, while narcissism emerged as the strongest predictor (β = 0.20, *p* < 0.01) of passive bystanderism, psychopathy did not significantly predict (*p* = 0.09) passive bystanderism (see Table 3). Notably, with the inclusion of DT traits, agreeableness ceased to be a significant predictor of both active trolling (*p* = 0.14) and passive bystanderism (*p* = 0.99). These results supported the premise that DT traits mediate the relationship between agreeableness and trolling behaviors.

### Mediation analyses

The mediating role of DT traits in the relationship between agreeableness and trolling dimensions was assessed using two separate SEMs. In the first model, active trolling was assessed as the dependent variable (SEM 1), and the second model focussed on passive bystanderism (SEM 2). Although Baron and Kenny^76^ recommend prefacing a full mediation model with a direct effect model, the absence of degrees of freedom precluded determining model fit for the direct effect. Furthermore, regression coefficients were previously established via hierarchical multiple regression analyses. Consequently, this study proceeded to test the hypothesized relationships directly using full mediation models in both SEM 1 and SEM 2. The mediation analyses employed a bias-corrected bootstrap estimation, with a specified bootstrap sample of 2000. Table 4 outlines the model pathways, which are considered significant if the 95% CIs do not encompass zero.

**Table 4:** Regression coefficients and model pathways (total, direct, and indirect effects) within SEM 1 and SEM 2. *N* = 497, β = standardized path coefficient, SE = standard error, 95% CI (BC) = 95% bias-corrected confidence intervals.

Model pathways	β	SE	95% CI (BC)	
			Lower	Upper
SEM 1: Agreeableness → Active Trolling				
Total effect	− 0.29	0.04	− 0.37	− 0.20
Direct effect	0.02	0.05	− 0.07	0.12
Indirect effect	− 0.31	0.04	− 0.40	− 0.24
SEM 2: Agreeableness → Passive Bystanderism				
Total effect	− 0.22	0.04	− 0.30	− 0.13
Direct effect	0.08	0.05	− 0.10	0.19
Indirect effect	− 0.30	0.04	− 0.40	− 0.23

### The mediating role of DT traits between agreeableness and active trolling

**Figure 2:**
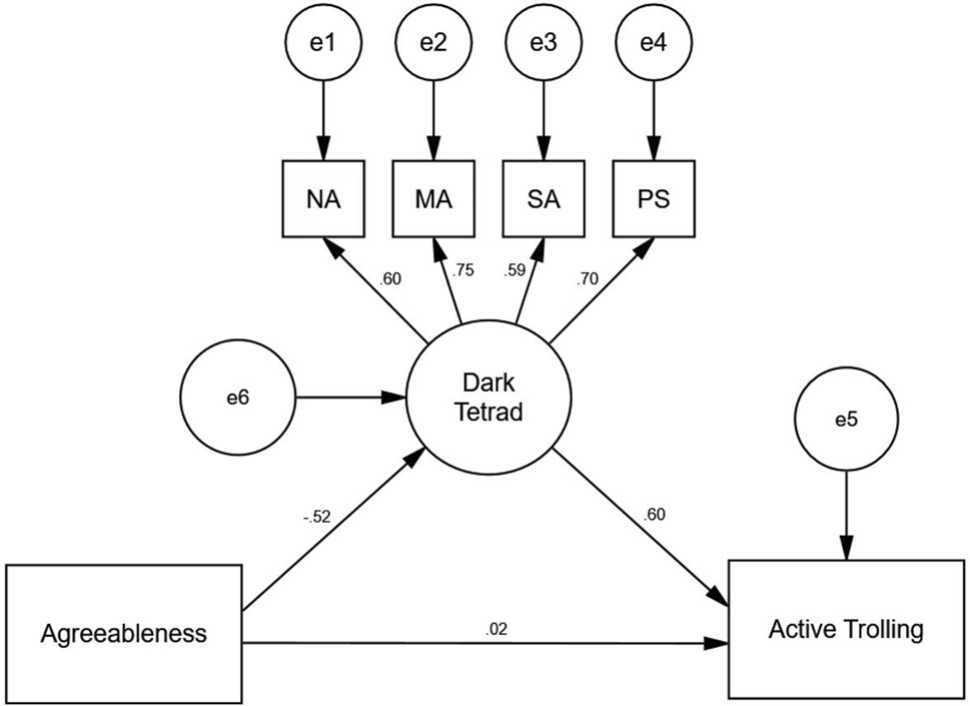
Standardized path coefficients of the proposed SEM 1, showing the mediating effect of DT on the relationship between agreeableness and active trolling. *N* = 497, χ^2^ = 25.25, *p* < 0.01, χ^2^/df = 3.15, CFI = 0.97, GFI = 0.97, RMSEA = 0.06. *NA* narcissism, *MA* Machiavellianism, *PS* psychopathy, *SA* sadism.

SEM 1 (see Fig. 2) examined the direct effect of agreeableness on active trolling and its indirect effect through DT traits. This model, reflecting the hypothesized relationships, indicated a good fit across a range of model fit indices (CFI = 0.97, GFI = 0.98, RMSEA = 0.06), with the exception of the Chi-square statistic (χ^2^ = 25.25, *p* < 0.01, χ^2^/df = 3.15).

The standardized path coefficients revealed that the indirect effect of agreeableness on active trolling (β = − 0.31, 95% CI = − 0.40 to − 0.24) was significant (see Table 4). However, with the inclusion of DT traits, there was no significant direct effect of agreeableness on active trolling (β = 0.02, 95% CI = − 0.07 to 0.12). The results supported hypothesis 3a, suggesting that DT traits fully mediated the relationship between agreeableness and active trolling.

### The mediating role of DT traits between agreeableness and passive bystanderism

**Figure 3:**
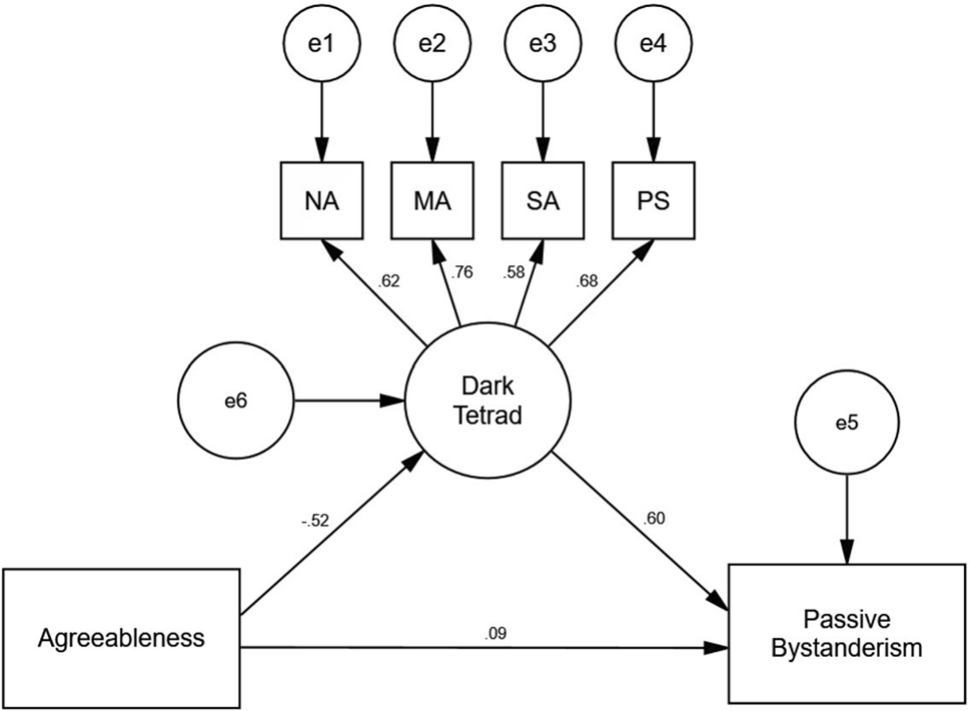
Standardized path coefficients of the proposed SEM 2, showing the mediating effect of DT on the relationship between agreeableness and passive bystanderism. *N* = 497, χ^2^ = 28.69, *p* < 0.01, χ^2^/df = 3.58, CFI = 0.97, GFI = 0.98, RMSEA = 0.06. *NA* narcissism, *MA* Machiavellianism, *PS* psychopathy, *SA* sadism.

Similar to SEM 1, the hypothesized relationships in SEM 2 (see Fig. 3) also displayed a strong fit across various model fit indices (CFI = 0.97, GFI = 0.98, RMSEA = 0.07), except for the Chi-square statistic (χ^2^ = 28.69, *p* < 0.01, χ^2^/df = 3.58).

The standardized path coefficients of SEM 2 indicated a significant indirect effect of agreeableness on passive bystanderism (β = − 0.30, 95% CI = − 0.40 to − 0.23). Yet, similar to SEM 1, including DT traits did not yield a significant direct effect of agreeableness on passive bystanderism (β = 0.08, 95% CI = − 0.10 to 0.19). Therefore, the results supported hypothesis 3b, suggesting that DT traits fully mediated the relationship between agreeableness and passive bystanderism, consistent with the results from SEM 1.

## Discussion

The present study extends the conventional perception of trolling as a unidimensional interaction, typically characterized by a perpetrator directly confronting a victim on SNSs. It proposed an alternative view where trolling can manifest through bystander presence, thus expanding the concept beyond the realm of direct attacks. Firstly, this study investigated active trolling and passive bystanderism by developing the GAATPB scale. Furthermore, it also examined how variations in certain personality traits are associated with the proposed trolling dimensions.

While the association and predictive utility of traits like agreeableness and DT for active trolling are well-documented, their role in influencing passive bystanderism remains unexplored. By addressing this research gap, the present study examined the relationship and predictive utility of agreeableness and DT traits on both trolling dimensions. It also investigated whether the lack of specific characteristics, such as agreeableness (lower agreeableness), might predispose individuals to exhibit DT traits, which could lead to subsequent trolling behaviors. Considering the overarching theme of reduced empathy across both trolling dimensions and the examined personality traits, the study hypothesized that no significant differences exist between active trolling and passive bystanderism in their relationships with personality traits (hypothesis 1), the predictive utility of these traits (hypothesis 2), and the assumed mediation of DT traits in the relationship between lower agreeableness and trolling dimensions (hypothesis 3). Building on these primary hypotheses, the study further investigated the role of DT traits and lower agreeableness separately on active trolling and passive bystanderism.

The results supported hypotheses 1a and 1b, demonstrating a significant relationship between the examined personality traits and trolling dimensions. This finding is in agreement with the previous studies^9,39,55^, where trolling was positively associated with all DT traits and negatively with agreeableness. The study highlights that the association of these personality traits with both forms of trolling is similar. Following the correlation, hierarchical multiple regression analyses were undertaken. Initially, the results showed that lower agreeableness predicted active trolling and passive bystanderism in the first step of the analyses. Yet, with the inclusion of DT traits in the second step, it ceased to be the predictor of both trolling dimensions, contradicting the previous research^1,34,39^. These findings indicate that lower agreeableness would no longer influence trolling behaviors when accounted for the shared variance with DT traits, suggesting a potential mediating role of DT traits in the dynamic between agreeableness and trolling behaviors.

Contrary to hypotheses 2a and 2b, narcissism and Machiavellianism emerged as significant predictors for active trolling and passive bystanderism, challenging the assertions from previous studies^9,34,39^. The current findings support the theory of threatened egotism^77^, which posits that cyber-aggressive behaviors, such as trolling, serve as a defense mechanism for narcissists to protect their favorable self-view against perceived threats. In SNSs, these aggressive behaviors often target individuals who oppose the narcissistic views or persona of the trolls^78,79^. Such aggression manifests as either active trolling, characterized by derogatory comments, or passive bystanderism, involving indirect reinforcement through actions like sharing content or following troll pages.

Mirroring the concept of threatened egotism, the role of narcissism in promoting trolling behaviors is further elucidated by the phenomenon of schadenfreude. This German term describes the experience of deriving pleasure from others’ misfortune and harboring desires for their adverse outcomes^80^. Schadenfreude is closely associated with increased levels of narcissism and the act of downward social comparison^81^, often driven by a need for self-enhancement. Consequently, individuals with lower self-esteem and negative self-perceptions are more prone to schadenfreude upon witnessing others’ failures through the act of trolling^82,83^. Narcissistic tendencies, such as schadenfreude, are primarily witnessed in the passive observation of others’ suffering, providing a subtler form of gratification that is considered illegitimate, given its lack of acquisition through direct competition^84^. Such a tendency is also reflected in the current findings, as narcissism emerged as the most robust predictor of passive bystanderism, which was devoid of prediction from a more callous psychopathic trait.

Similarly, individuals with pronounced Machiavellian tendencies often employ manipulation tactics, such as inducing feelings of shame or guilt, in their online social interactions^85^. Rauthmann^86^ describes this as protective self-monitoring, where Machiavellian individuals continually adjust their behavior for social advantage and control. In the context of internet trolling, these tendencies can lead to subtle forms of manipulation, such as gaslighting or disseminating misinformation. The immediacy and anonymity of SNSs can amplify active trolling and passive bystanderism, providing a platform for individuals with high levels of narcissism and Machiavellianism to assert dominance or manipulate others without apparent consequences.

Interestingly, although the study did not formulate specific hypotheses, the results revealed a differential impact of psychopathy across trolling dimensions. While psychopathy emerged as the strongest predictor of active trolling, it did not predict passive bystanderism. This finding aligns with the notion that individuals with high psychopathic tendencies are attracted to the excitement of causing online disruptions, consistent with their thrill-seeking tendencies^14^. Additionally, the deceptive nature of active trolling harmonizes with the callous and unemotional traits typically seen in psychopathy, along with their manipulative interpersonal style^87^. The bullying behavior, regardless of whether it transpires offline or online, will keep psychopaths motivated and committed to their impulsive ideas as it makes them feel good to instigate distress in others.

However, the findings reflect that being a passive bystander to the troll and not participating in it does not support callous and unemotional impulsivity; hence, it is uncertain that such an individual possesses psychopathic tendencies. Although closely related to psychopathy, trait sadism was found to be a predictor of both active trolling and passive bystanderism, corroborating previous studies^1,9^. Individuals who engage in active trolling on SNSs often taunt and humiliate others, actively seeking such opportunities^63^. Moreover, those with pronounced sadistic traits are characterized not only by their direct involvement in online aggression but also by deriving pleasure from observing and endorsing such behaviors. The study suggests that while passive bystander trolls may not exhibit overt psychopathic traits, the presence of sadistic tendencies could contribute to their engagement in and reinforcement of trolling behaviors.

The results from the mediation analyses revealed that DT traits fully mediated the relationship between lower agreeableness and both active trolling, as well as passive bystanderism, thereby affirming hypotheses 3a and 3b, respectively. Individuals characterized by lower agreeableness tend to prioritize their own needs, engage in manipulative behaviors, and exhibit aggressive tendencies^88,89^. Crucially, they show a significant lack of empathy towards others^90^. Studies on empathy^45,59,91^ indicate that while the DT exhibits some correlation with empathic concern, it does not significantly predict a lack of empathy beyond the influence of lower agreeableness. These findings imply that a certain degree of cognitive apathy, fueled by lower agreeableness, might be necessary for engaging in harmful behaviors like trolling. This inherent lack of empathy and increased likelihood of adversarial behavior might also predispose those with lower agreeableness to adopt passive bystander roles. Rather than intervening or offering support to victims, their reduced empathetic engagement and propensity for contentiousness might lead them to either ignore the distress of others or derive satisfaction from observing conflict without direct participation. Such passive bystander behavior can thus be seen as an extension of their broader interpersonal conduct, where a deficiency in positive social engagement and a predisposition towards antagonism influence their actions (or inactions) within both direct and vicarious social interactions. Therefore, it is logical to assert low agreeableness as a common denominator underlying dark personality traits and both active trolling and passive bystanderism. Consequently, the study emphasizes that trolling behavior stems not merely from lower agreeableness but also from a lack of consensus, triggered by the activation of inherent dark traits.

The issue of lower agreeableness and manifestations of dark personalities hold significant implications for online mental health, impacting not only the individuals exhibiting these traits but also their victims and society at large. Considering this, lower agreeableness and dark personalities warrant increased research attention. This is particularly crucial in understanding their correlation and influence on internet trolling, a growing concern in the digital age. There remains much to explore about the underlying processes and factors associated with these personality traits and their role in fostering a range of online deviant behaviors, which can further exacerbate the negative impacts on individual and societal mental health.

While this study contributes to the understanding of bystander behavior in trolling literature, it is not without limitations. One primary limitation is the scope of the GAATPB scale developed for this study. The scale did not encompass the multidimensionality of bystander trolling, suggesting that future research should broaden this scope to include positive bystander interventions. Additionally, the survey research method provided valuable insights but did not deeply explore the phenomenological aspects of trolling, such as subjective experiences, motivations, and emotions. Future studies should incorporate methodologies that probe these subjective dimensions. Another limitation is the cumulative assessment of dark personality traits. A more nuanced exploration of these traits in a continuum (such as primary and secondary psychopathy) could yield deeper insights into the underlying mechanisms of trolling behavior^55^. The study also suggests a more granular approach to examining trolling within specific domains of SNSs, acknowledging that trolling behaviors may vary substantially across different online platforms, such as Facebook or dating apps. Lastly, the study recognizes the potential impact of target attributes on trolling behaviors^35^. Future research incorporating these elements could offer a more holistic view of both trolls and their targets, enhancing the understanding of trolling dynamics.

## Conclusion

This study investigated active trolling and passive bystanderism and their relationship with personality traits by developing the GAATPB scale. The investigators examined how DT traits and agreeableness correlate with and predict these trolling behaviors. The findings showed a significant relationship between DT traits and agreeableness with active trolling and passive bystanderism, highlighting a shared psychological basis for these behaviors. Notably, while psychopathy emerged as the strongest predictor for active trolling, it did not predict passive bystanderism. In contrast, trait sadism was a consistent predictor for both, emphasizing its role in online misconduct. This study also challenged previous notions by demonstrating that narcissism and Machiavellianism significantly predicted trolling behaviors. Furthermore, the findings indicate that trolling behavior, while stemming from lower agreeableness, is effectively mediated by the DT traits.

### Supplementary Information

Supplementary Information.

## Data Availability

The associated data can be requested by contacting the corresponding author.
