# Supplementary material for: The differential effect of psychopathy on active and bystander trolling behaviors: the role of dark tetrad traits and lower agreeableness
Source: Sci Rep. 2024 Apr 30;14:9905. doi: 10.1038/s41598-024-60203-6 (PMC11061140; doi:10.1038/s41598-024-60203-6)
Supplement: Supplementary file 1 — Supplementary Information. [file 41598_2024_60203_MOESM1_ESM.docx]

**Supplementary Information**

**The Differential Effect of Psychopathy on Active and Bystander Trolling Behaviors: The Role of Dark Tetrad Traits and Lower Agreeableness**

**Anantha Ubaradka^1,2^, and Sanjram Premjit Khanganba^1,2,*^**

^1^Human Factors & Applied Cognition Lab, Indian Institute of Technology Indore, Indore, 453552, India

^2^Discipline of Psychology, Indian Institute of Technology Indore, Indore, 453552, India

^*^ sanjrampk@iiti.ac.in, sanjrampk@gmail.com

**Assumptions of the Regression Analyses: Active Trolling**

This study meticulously confirmed the assumptions required for regression analyses, ensuring the integrity and reliability of the findings. Supplementary Table 1 details the variance changes when predictors are introduced at different stages of the analyses, alongside the Durbin-Watson value. This value indicates a satisfactory level of autocorrelation among the residuals, with a value of 2 suggesting no autocorrelation and values below 2 indicating positive autocorrelation, thereby demonstrating minimal to nil autocorrelation in this case.

| Model | R | R Square | Change Statistics | | | | | **Durbin-Watson** |  |
| --- | --- | --- | --- | --- | --- | --- | --- | --- | --- |
|  |  |  | R Square Change | F Change | df1 | df2 | Sig. F Change |  |  |
| 1 | 0.294^a^ | 0.086 | 0.086 | 46.745 | 1 | 495 | 0.000 |  |  |
| 2 | 0.526^b^ | 0.277 | 0.191 | 32.351 | 4 | 491 | 0.000 | **2.045** |  |
| **Supplementary Table 1.** Model summary.  a. Predictors: (Constant), Agreeableness | | | | | | | | | |
| b. Predictors: (Constant), Agreeableness, Narcissism, Sadism, Psychopathy, Machiavellianism | | | | | | | | | |
|  | | | | | | | | | |

The regression coefficients are displayed in Supplementary Table 2, which includes a collinearity analysis, revealing no concerns about collinearity. This is evidenced by all Tolerance values being below 1 and the VIF not exceeding 10, indicating that the predictors maintain their distinctiveness without unduly influencing each other.

| Model | | Standardized Coefficients | t | Sig. | 95.0% Confidence Interval for B | | **Collinearity Statistics** | |
| --- | --- | --- | --- | --- | --- | --- | --- | --- |
|  |  | Beta |  |  | Lower Bound | Upper Bound | **Tolerance** | **VIF** |
| 1 | (Constant) |  | 14.857 | 0.000 | 12.148 | 15.850 |  |  |
|  | Agreeableness | -0.294 | -6.837 | 0.000 | -0.243 | -0.135 | **1.000** | **1.000** |
| 2 | (Constant) |  | 2.276 | 0.023 | 0.408 | 5.554 |  |  |
|  | Agreeableness | -0.064 | -1.477 | 0.140 | -0.096 | 0.014 | **0.778** | **1.285** |
|  | Sadism | 0.168 | 3.750 | 0.000 | 0.054 | 0.172 | **0.731** | **1.367** |
|  | Machiavellianism | 0.179 | 3.548 | 0.000 | 0.070 | 0.243 | **0.579** | **1.727** |
|  | Narcissism | 0.108 | 2.353 | 0.019 | 0.014 | 0.157 | **0.702** | **1.425** |
|  | Psychopathy | 0.192 | 4.001 | 0.000 | 0.094 | 0.275 | **0.643** | **1.556** |

**Supplementary Table 2.** Regression coefficients and collinearity statistics.

Furthermore, Supplementary Table 3 shows whether outliers exist within the dataset. Given that the standard residuals do not exceed a value of 3.29, it confirms the absence of outliers in the data presented. This meticulous approach to verifying statistical assumptions ensures the study’s conclusions are based on robust and reliable analytical procedures.

|  | Minimum | Maximum | Mean | Std. Deviation | N |
| --- | --- | --- | --- | --- | --- |
| Predicted Value | 4.3247 | 11.7281 | 7.6137 | 1.53368 | 497 |
| Residual | -5.85698 | 7.90161 | 0.00000 | 2.47860 | 497 |
| Std. Predicted Value | -2.144 | 2.683 | 0.000 | 1.000 | 497 |
| Std. Residual | **-2.351** | **3.172** | 0.000 | 0.995 | 497 |

**Supplementary Table 3.** Residual statistics.

The histogram (see Supplementary Fig. 1) and the P-P plot (see Supplementary Fig. 2) illustrates the normal distribution of the standardized residuals. Subsequently, the partial regression plots (see Supplementary Fig. 3 to Supplementary Fig. 7) show the homoscedasticity of the dataset for all the predictors of active trolling.


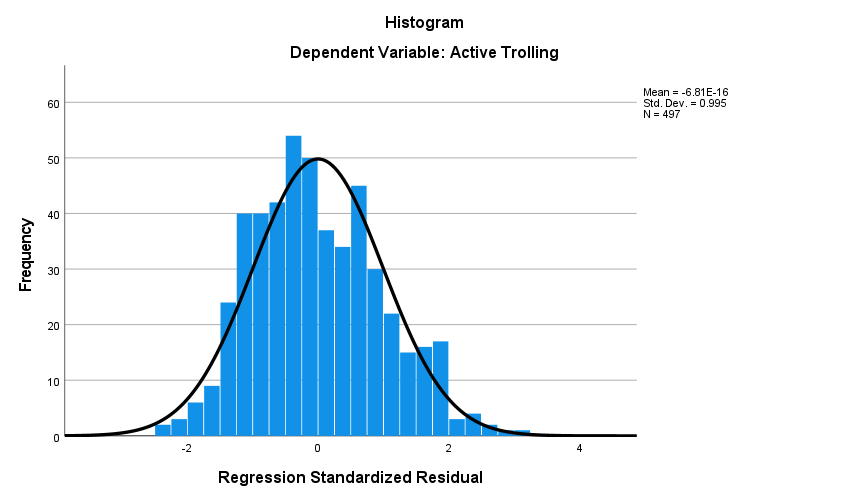


**Supplementary Figure 1.** Normal distribution of the standardized residuals.


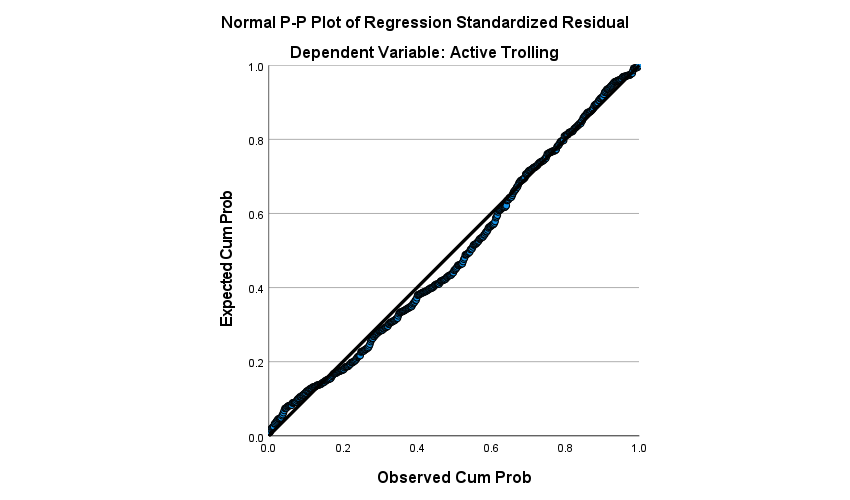


**Supplementary Figure 2.** Normal P-P plot of regressions standardized residuals.


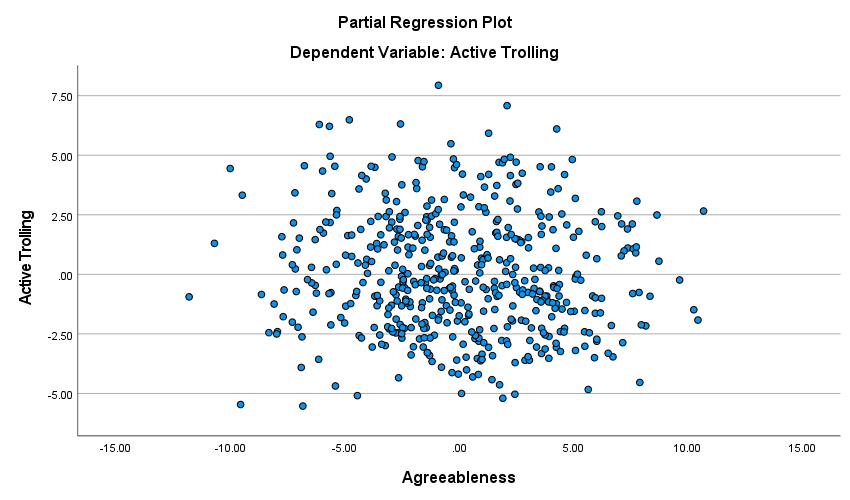


**Supplementary Figure 3.** Partial regression plot of agreeableness on active trolling.


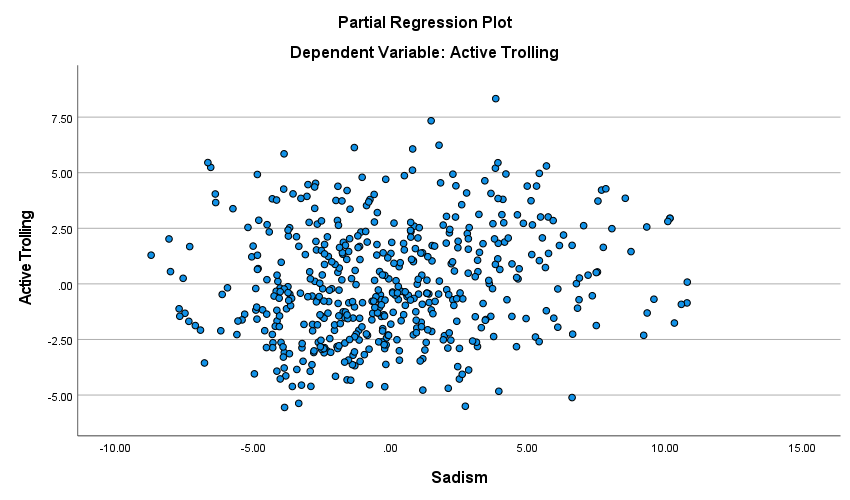


**Supplementary Figure 4.** Partial regression plot of sadism on active trolling.


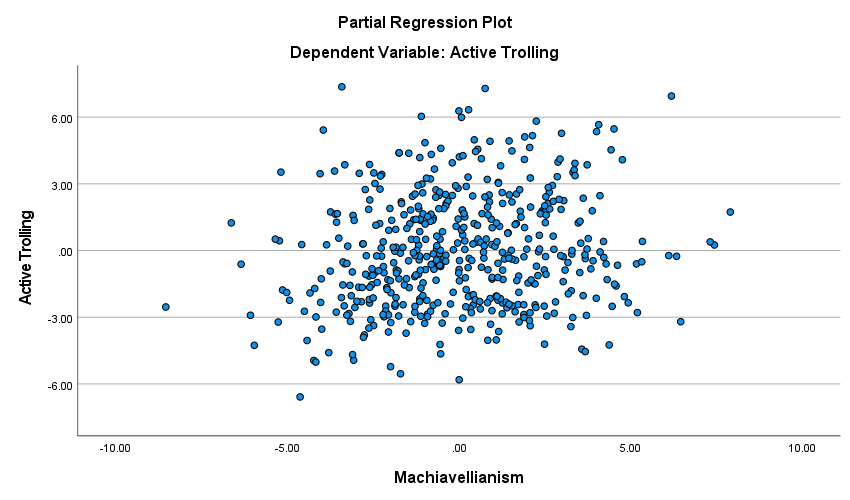


**Supplementary Figure 5.** Partial regression plot of Machiavellianism on active trolling.


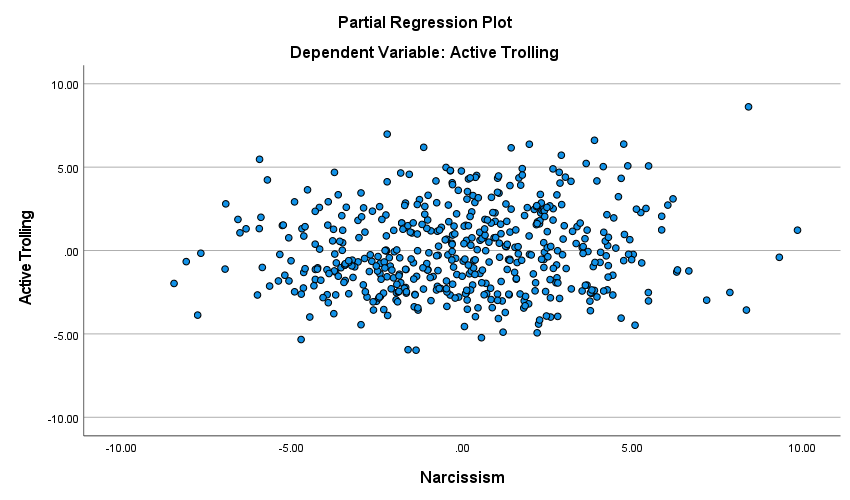


**Supplementary Figure 6.** Partial regression plot of narcissism on active trolling.


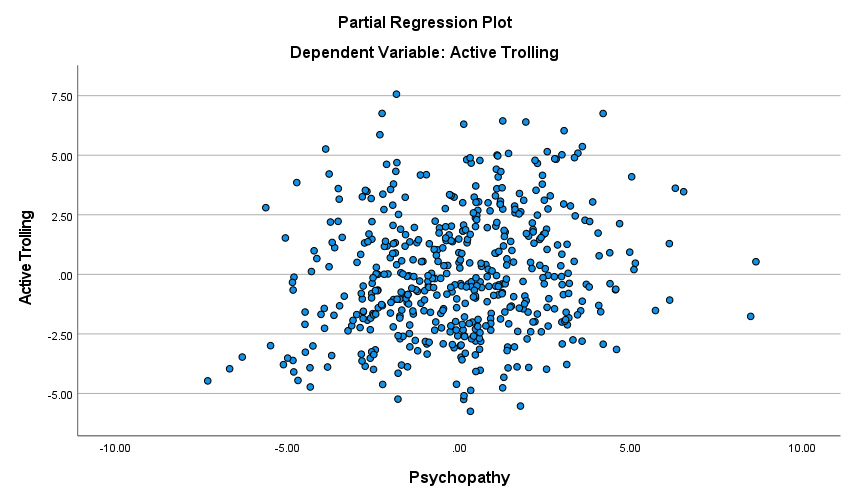


**Supplementary Figure 7.** Partial regression plot of psychopathy on active trolling.

**Assumptions of the Regression Analysis: Passive Bystanderism**

Supplementary Table 4 details the variance changes when predictors are introduced at different stages of the analyses alongside the Durbin-Watson value. This value indicates a satisfactory level of autocorrelation among the residuals. As the Durbin-Watson value is marginally below 2, it can be concluded that no autocorrelation or slightly positive correlation exists between the variables.

| Model | R | R Square | Change Statistics | | | | | **Durbin-Watson** |  |
| --- | --- | --- | --- | --- | --- | --- | --- | --- | --- |
|  |  |  | R Square Change | F Change | df1 | df2 | Sig. F Change |  |  |
| 1 | .220^a^ | 0.048 | 0.048 | 25.094 | 1 | 495 | 0.000 |  |  |
| 2 | .491^b^ | 0.241 | 0.193 | 31.224 | 4 | 491 | 0.000 | **1.934** |  |
| **Supplementary Table 4.** Model summary.  a. Predictors: (Constant), Agreeableness | | | | | | | | | |
| b. Predictors: (Constant), Agreeableness, Narcissism, Sadism, Psychopathy, Machiavellianism | | | | | | | | | |
| c. Dependent Variable: Passive Bystanderism | | | | | | | | | |

Supplementary Table 5 elaborates on the regression coefficients and includes a collinearity analysis, revealing no collinearity concerns. This is evidenced by all Tolerance values being below 1 and the VIF not exceeding 10, indicating that the predictors maintain their distinctiveness without unduly influencing each other.

| Model | | Standardized Coefficients | t | Sig. | 95.0% Confidence Interval for B | | **Collinearity Statistics** | |
| --- | --- | --- | --- | --- | --- | --- | --- | --- |
|  |  | Beta |  |  | Lower Bound | Upper Bound | **Tolerance** | **VIF** |
| 1 | (Constant) |  | 12.623 | 0.000 | 10.594 | 14.500 |  |  |
|  | Agreeableness | -0.220 | -5.009 | 0.000 | -0.203 | -0.089 | **1.000** | **1.000** |
| 2 | (Constant) |  | 0.997 | 0.319 | -1.342 | 4.107 |  |  |
|  | Agreeableness | 0.000 | 0.005 | 0.996 | -0.058 | 0.058 | **0.778** | **1.285** |
|  | Sadism | 0.168 | 3.653 | 0.000 | 0.054 | 0.179 | **0.731** | **1.367** |
|  | Machiavellianism | 0.194 | 3.761 | 0.000 | 0.084 | 0.268 | **0.579** | **1.727** |
|  | Narcissism | 0.199 | 4.251 | 0.000 | 0.088 | 0.239 | **0.702** | **1.425** |
|  | Psychopathy | 0.083 | 1.689 | 0.092 | -0.013 | 0.178 | **0.643** | **1.556** |

**Supplementary Table 5.** Regression coefficients and collinearity statistics.

Furthermore, Supplementary Table 6 shows whether any outliers exist within the dataset. Given that the standard residuals do not exceed a value of 3.29, it confirms the absence of outliers in the data presented. This meticulous approach to verifying statistical assumptions ensures the study’s conclusions are based on robust and reliable analytical procedures.

|  | Minimum | Maximum | Mean | Std. Deviation | N |
| --- | --- | --- | --- | --- | --- |
| Predicted Value | 4.2372 | 11.6924 | 7.6117 | 1.47979 | 497 |
| Residual | -7.38256 | 6.44760 | 0.00000 | 2.62430 | 497 |
| Std. Predicted Value | -2.280 | 2.758 | 0.000 | 1.000 | 497 |
| Std. Residual | **-2.799** | **2.444** | 0.000 | 0.995 | 497 |

**Supplementary Table 6.** Residual statistics.

Furthermore, the histogram (see Supplementary Fig. 8) and the P-P plot (see Supplementary Fig. 9) illustrate the normal distribution of the standardized residuals. Subsequently, the partial regression plots (see Supplementary Fig. 10 to Supplementary Fig. 14) show the homoscedasticity of the dataset for all the predictors of passive bystanderism.


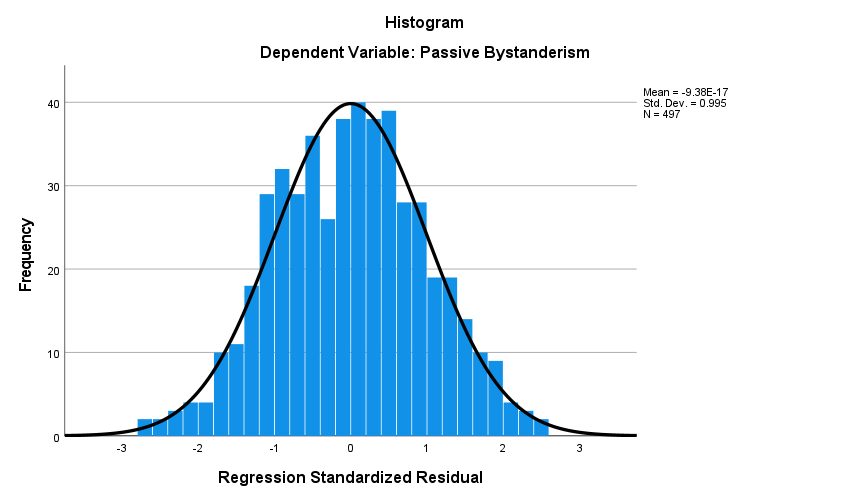


**Supplementary Figure 8.** Normal distribution of the standardized residuals.


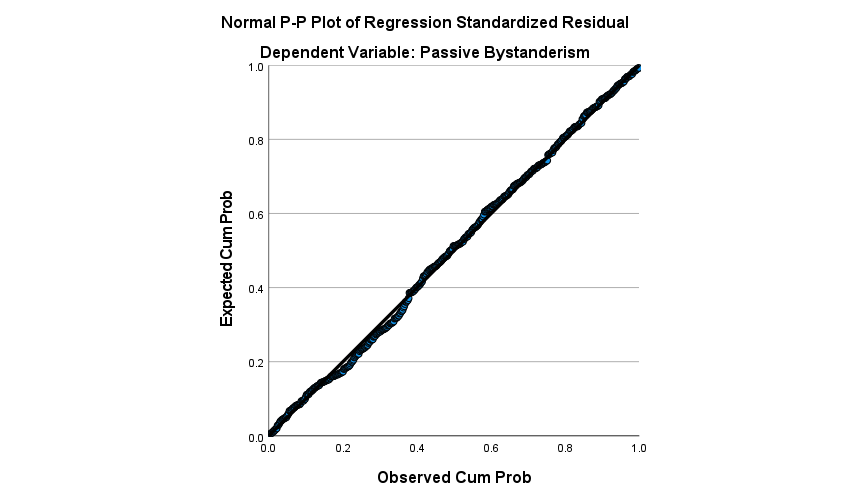


**Supplementary Figure 9.** Normal P-P plot of regressions standardized residuals.


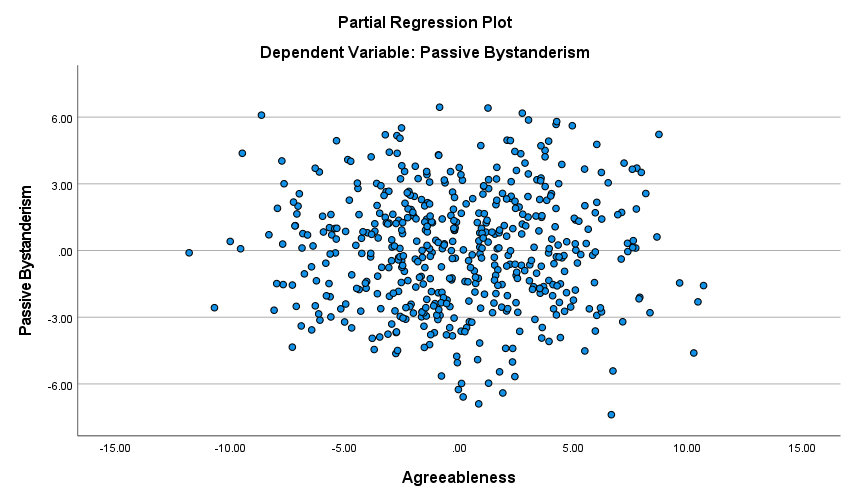


**Supplementary Figure 10.** Partial regression plot of agreeableness on passive bystanderism.


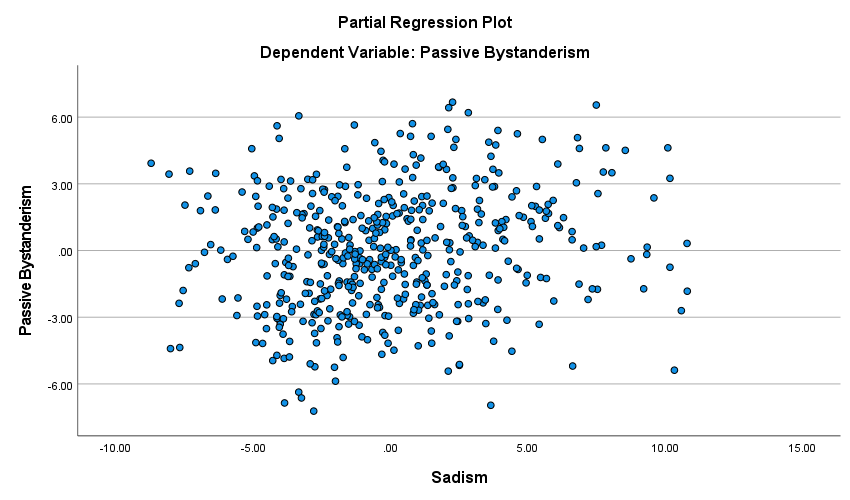


**Supplementary Figure 11.** Partial regression plot of sadism on passive bystanderism.


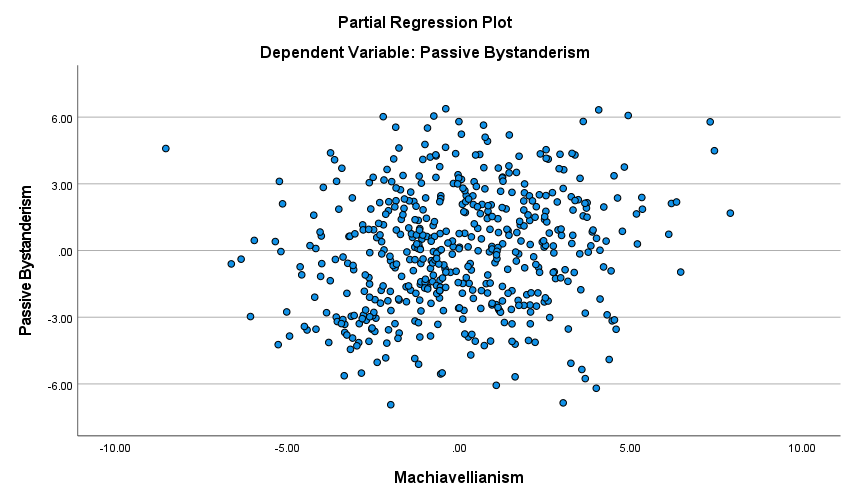


**Supplementary Figure 12.** Partial regression plot of Machiavellianism on passive bystanderism.


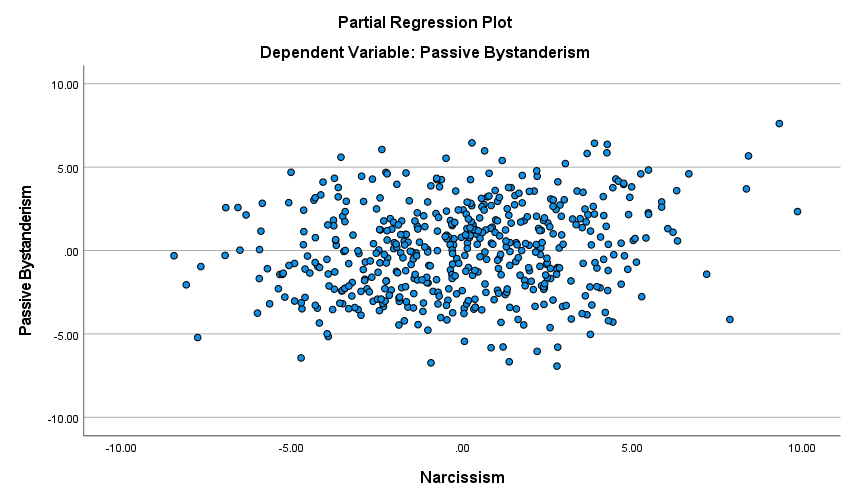


**Supplementary Figure 13.** Partial regression plot of narcissism on passive bystanderism.


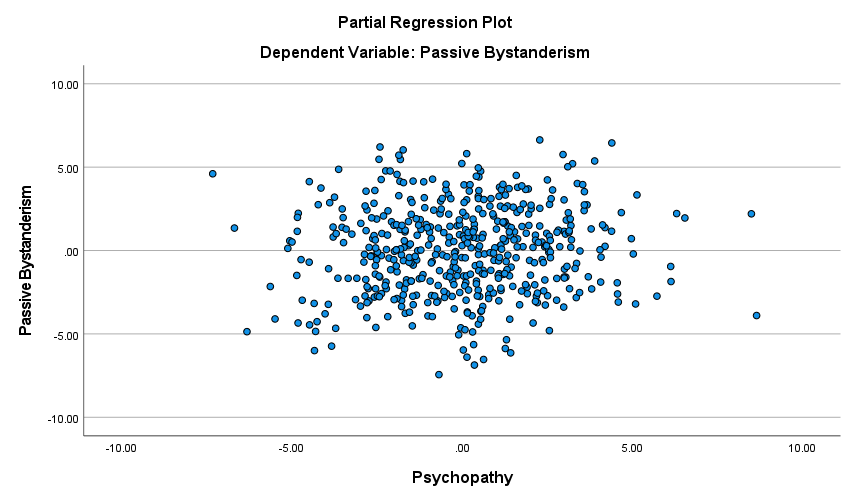


**Supplementary Figure 14.** Partial regression plot of psychopathy on passive bystanderism.
